# Mitochondrial dysfunction in microglia: a novel perspective for pathogenesis of Alzheimer’s disease

**DOI:** 10.1186/s12974-022-02613-9

**Published:** 2022-10-06

**Authors:** Yun Li, Xiaohuan Xia, Yi Wang, Jialin C. Zheng

**Affiliations:** 1 Center for Translational Neurodegeneration and Regenerative Therapy, Tongji Hospital Affiliated to Tongji University School of Medicine, Shanghai, 200072 China; 2 Shanghai Frontiers Science Center of Nanocatalytic Medicine, Shanghai, 200331 China; 3 Key Laboratory of Spine and Spinal Cord Injury Repair and Regeneration (Tongji University), Ministry of Education, Shanghai, 200065 China; 4 Translational Research Institute of Brain and Brain-Like Intelligence, Shanghai Fourth People’s Hospital affiliated to Tongji University School of Medicine, Shanghai, 200434 China; 5 Translational Research Center, Shanghai Yangzhi Rehabilitation Hospital Affiliated to Tongji University School of Medicine, Shanghai, 201613 China; 6 Collaborative Innovation Center for Brain Science, Tongji University, Shanghai, 200092 China

**Keywords:** Microglia, Alzheimer’s disease, Mitochondria, Metabolism, mtDNA

## Abstract

Alzheimer's disease (AD) is the most common neurodegenerative disease in the elderly globally. Emerging evidence has demonstrated microglia-driven neuroinflammation as a key contributor to the onset and progression of AD, however, the mechanisms that mediate neuroinflammation remain largely unknown. Recent studies have suggested mitochondrial dysfunction including mitochondrial DNA (mtDNA) damage, metabolic defects, and quality control (QC) disorders precedes microglial activation and subsequent neuroinflammation. Therefore, an in-depth understanding of the relationship between mitochondrial dysfunction and microglial activation in AD is important to unveil the pathogenesis of AD and develop effective approaches for early AD diagnosis and treatment. In this review, we summarized current progress in the roles of mtDNA, mitochondrial metabolism, mitochondrial QC changes in microglial activation in AD, and provide comprehensive thoughts for targeting microglial mitochondria as potential therapeutic strategies of AD.

## Background

Alzheimer's disease (AD) is one of the most prevalent neurodegenerative diseases and the No. 1 cause of dementia in the world [1, 2]. With the aging of global population, the prevalence and incidence of AD keep increasing [1–4]. By 2050, the prevalence of dementia will triple worldwide, putting a tremendous emotional and financial load on society [1]. AD is mainly manifested as progressive and selective loss of neurons, resulting in memory impairment and executive dysfunction, accompanied by neuropsychiatric symptoms. The main pathological features of AD include extracellular β-amyloid (Aβ) plaques and intracellular neurofibrillary tangles (NFTs) composed of phosphorylated Tau protein (p-Tau) [5, 6]. The complex Aβ–Tau interaction has synergistic effect on the pathogenesis of AD [7]. However, clinical trials that aim to target Aβ and NFTs fail to obtain satisfied results, suggesting the necessity to investigate AD pathogenesis in a novel perspective [8].

Microglia are innate immune cells in the central nervous system (CNS) and play an important role in the phagocytosis and clearance of pathogenic molecules [9–11] and neuroinflammatory responses [12]. Mounting evidence has shown that microglia play a critical role in Aβ and p-Tau-mediated neuronal dysfunction in the pathogenesis of AD [13, 14]. Aβ has been found to interact with the receptor for advanced glycation end products (RAGE) in microglia, which may result in excessive activation of microglia and the production of pro-inflammatory molecules [11, 13, 15].

Proinflammatory microglia substantially alter brain energy metabolism and ROS/NO production, leading to impairment of neural network function, neurodegeneration, and blood–brain barrier dysfunction that contribute to the pathogenesis of AD [12, 16, 17]. Hence, microglia perform a variety of specialized functions in AD, including phagocytosis and clearance of Aβ and p-Tau proteins, removal of dying neurons, and release of neurotrophic factors that support neuronal cells [5, 18]. These immune functions demand high energy, which are regulated by mitochondria.

Mitochondria are maternally inherited organelles that play critical roles in oxidative stress, energy metabolism, calcium homeostasis, and cell survival [19]. AD has been proposed as a metabolic disease [18–21]. Importantly, a strong correlation between microglial activation and metabolic dysfunction in AD has been demonstrated in both basic research and clinical studies [5, 18, 19]. In AD, a series of mitochondrial abnormalities have been identified, including structure alteration, age-dependent accumulation of mitochondrial DNA (mtDNA) changes, altered mitochondrial membrane potential, excessive mitochondrial ROS production, reduced mitochondrial adenosine triphosphate (ATP), disrupted electron transport chain (ETC), and increased mitochondrial fragmentation, leading to defective mitophagy in microglia and other brain cells [13, 22–29]. In addition, chronic exposure to Aβ and p-Tau induces dysregulated expression of late-onset AD-associated genes [30], mitochondrial toxicity, and metabolic dysfunction in microglia [31]. Moreover, therapies targeting basic mitochondrial processes, such as quality control (QC), show great therapeutic potential [32]. This review aims to summarize the investigations on the association of mitochondrial dysfunction with the inflammatory responses and the phagocytic capacity of microglia, and its involvement in the onset and progression of AD. Future directions that deepen our understanding of mitochondrial dysfunction in AD and facilitate the development of novel therapeutic strategies were also provided.

## mtDNA

### Structure and properties of mtDNA

Mitochondria is the only organelle in mammalian cells with genetic effects outside nucleus, with their unique circular genome called mtDNA. Human mtDNA contains 16,569 base pairs and has 37 coding genes, including 22 tRNAs, 2 rRNAs and 13 polypeptides (ETC complex I, complex III, complex IV and complex V) [33].

It is viewed as multiple copies, with each mitochondrion containing 2 to 10 DNA molecules and thousands to hundreds of thousands of copies in the cell. mtDNA consists of a heavy H chain, a light L chain, and a non-coding control region (D-loop) that tends to coil into nucleoid structures similar to bacterial DNA. Due to the lack of histone protection, the activity of DNA repair enzymes is also limited, making mtDNA extremely vulnerable to various damages, particularly reactive oxygen species (ROS) generated nearby. As a result, mitochondrial genes are 10 times more likely to mutate than nuclear genes [33]. Each cell contains hundreds of mitochondria, resulting in the coexistence of mutant and wild-type mtDNA, a condition known as heterogeneity [34]. The integrity of mtDNA is required for proper energy supply of mitochondria, as it encodes several subunits of mitochondrial respiratory chain complex as well as other mitochondrial proteins [33]. Previous studies show that mtDNA mutations are associated with the pathogenesis of neurological diseases, including neurodegenerative diseases [33, 35].

### mtDNA and AD

Growing evidence has suggested the association of mtDNA abnormality with AD pathogenesis [26, 27, 33]. Clinical studies found that mtDNA levels in the frontal cortex of AD patients were reduced by 30–50% compared with controls [36, 37]. A comprehensive in-depth analysis of more than 1000 human brain mtDNA sequence variants and abundances further confirmed the association between mtDNA deletion and AD [38]. For instance, a near-study-wide significant result was observed in AD patients with a deleterious MT-ND4L Asp88Glu missense mutation [28]. The mitochondrial-wide association study (MiWAS) also identified a mitochondrial single nucleotide polymorphism (SNP) rs2853499 that is associated with AD [39]. Characterization of mitochondrial DNA copy number (mtDNAcn) in 1361 aged brain samples revealed a 7–14% reduction of mtDNAcn in AD patients compared with controls, which is directly related to poor cognitive function [40]. More importantly, Swerdlow et al. analyzed mtDNA haplogroup frequencies in mtDNA-sequenced subjects of the Alzheimer’s disease Neuroimaging Initiative cohort in Kansas, USA, and reported that inherited mtDNA variants significantly influence AD risk, further indicating the necessity to focus on mtDNA regarding AD pathogenesis [41].

Interestingly, increased 5-methylcytosine levels are found in the D-loop region of mtDNA in the entorhinal cortex in brain samples of AD patients [42]. Moreover, dynamic DNA methylation patterns in the D-loop have been observed in the cerebral cortex of AD transgenic (APP/PS1) mice along AD pathology progression. Animal studies further demonstrated that mtDNA modifies learning, exploration, sensory development and the anatomy of mouse brains, providing direct evidence of the mtDNA mutation/deletion-induced cognitive impairment [43]. To further investigate the involvement of mtDNAs in AD, cytoplasmic hybrid (cybrid) cells were made by introducing platelet mitochondria of sporadic AD subjects into replicating clonal host cells that are deprived of their own mtDNA (ρ^0^ cells) [44, 45]. mtDNA from AD patients significantly decreased COX activity and increased ROS generation and Aβ deposition in cybrid cells [44, 45]. Hence, mtDNA abnormality and damage are tightly associated with the onset and progression of AD.

### Microglial mtDNA and AD

Given the important pathological roles of microglia, the mutation or deletion of microglial mtDNA has been reported to be involved in AD and other neurological disorders including cerebral ischemia/reperfusion [46, 47]. A recent study assessed microglial mtDNA depletion in the human AD brain and identified probable correlations between microglial mtDNA depletion levels in three brain regions with different AD pathological susceptibility and corresponding disease stages [48]. Continuous decrease of mtDNA deletion was observed in microglia in the hippocampus, a highly susceptible region to AD changes, while a persistent but slight increase of mtDNA deletion was found in microglia in the cerebellum during the progression of the disease [48]. This finding implies microglial mtDNA abnormality as an early event of AD.

Microglial mtDNA abnormality contributes to microglial dysfunctions in various pathological conditions. Studies performed on congenic mtDNA mice demonstrated the direct regulation of microglial mtDNA polymorphisms on the activation and cerebral Aβ phagocytosis of microglia [46]. The release of mtDNA from damaged cells could be sensed from microglia through different pathways including Toll-like receptor 9 (TLR9), cytosolic cyclic GMP-AMP synthase (cGAS)-stimulator of interferon genes (STING), and NOD-like receptor family pyrin domain containing 3 (NLRP3) inflammasomes [49]. Oxidized mtDNA binds to TLR9 present on the endo-lysosomal membrane and cause the activation of NLRP3 inflammasomes and IFN-related pathway [49]. After being internalized by nearby microglia, mtDNA binds to cGAS to activate the cytosolic DNA-sensing cGAS-STING pathway, thereby enhancing the expression of IFN-β [47]. Moreover, mtDNA-treated human microglia produce a great amount of ROS to activate Nf-κB signaling pathway and synthesize excessive pro-inflammatory cytokines, aggravating the inflammatory microenvironment to induce cell death and tissue damage [50]. Consequently, the release of damage-associated molecular patterns (DAMPs) is further enhanced, which, in turn, activates more microglia to propagate a vicious cycle of neuroinflammation [49, 51]. These observations implicate microglial mtDNA abnormality as a causal factor of glial activation and deposition of pathogenic substances in the pathogenesis of AD.

**Fig. 1:**
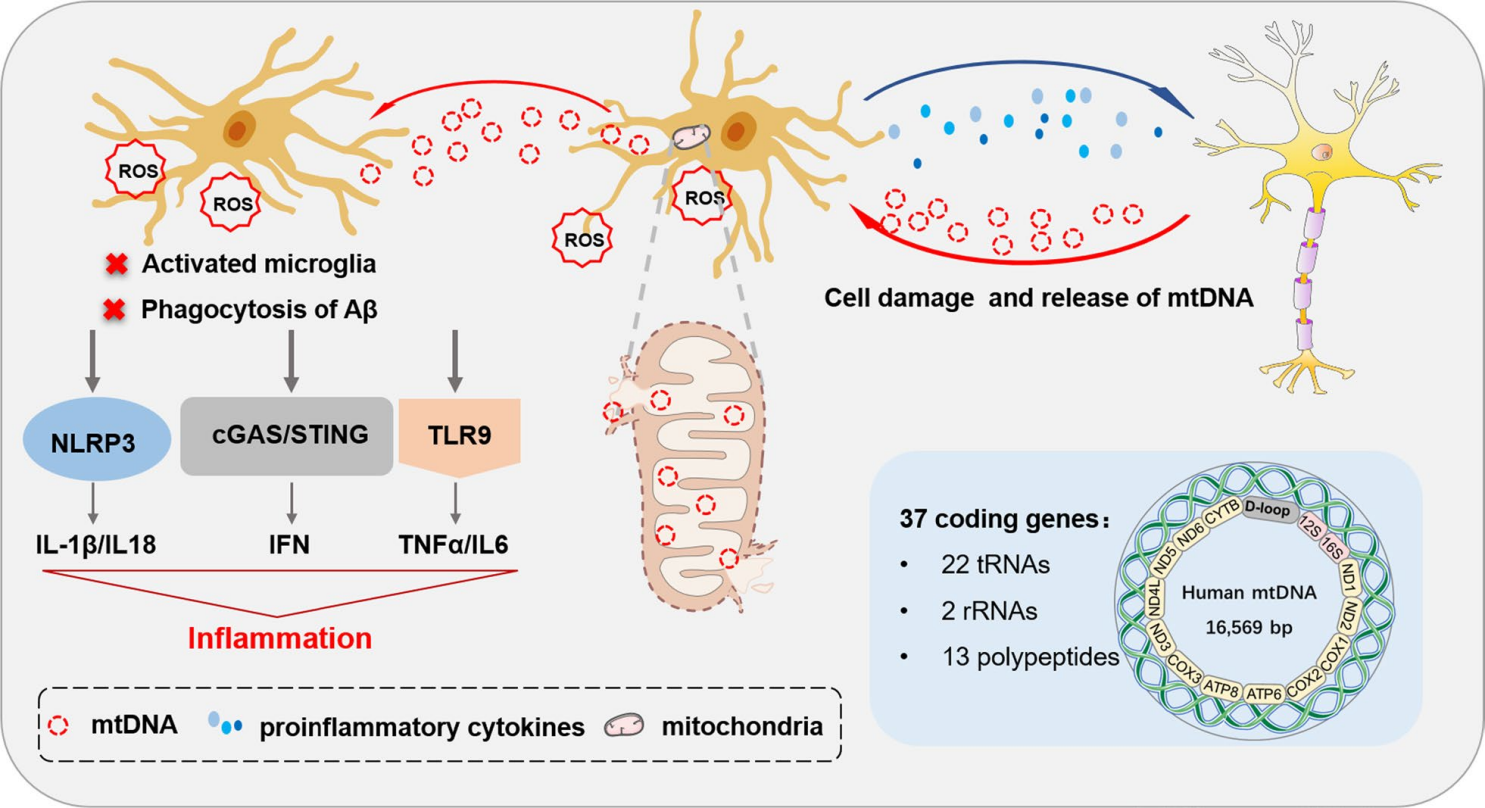
Microglial mtDNA in AD. Mitochondria has their unique circular genome called mtDNA. Human mtDNA contains 16,569 base pairs and 37 coding genes, including 22 tRNAs, 2 rRNAs and 13 polypeptides. In AD, microglial mtDNAs are vulnerable to various damages, particularly ROS generated nearby. Damaged mitochondria release abnormal mtDNA to adjacent microglia, which exacerbates inflammatory responses of microglia by triggering TLR and IFN expression and NLRP3 inflammasome activation, amplifying inflammatory effects. Moreover, activated microglia release pro-inflammatory cytokine to neurons, inducing mitochondrial dysfunction and irreversible neuronal damage. Damaged neurons further release mtDNA to microglia, creating a vicious cycle.

Taken together, mtDNA abnormality triggers mitochondrial dysfunction, causing inflammatory responses and microglial activation and ultimately leading to irreversible neuronal death and loss of glial function (Fig. 1).

## Mitochondrial metabolism

### Mitochondrial energy metabolism

**Fig. 2:**
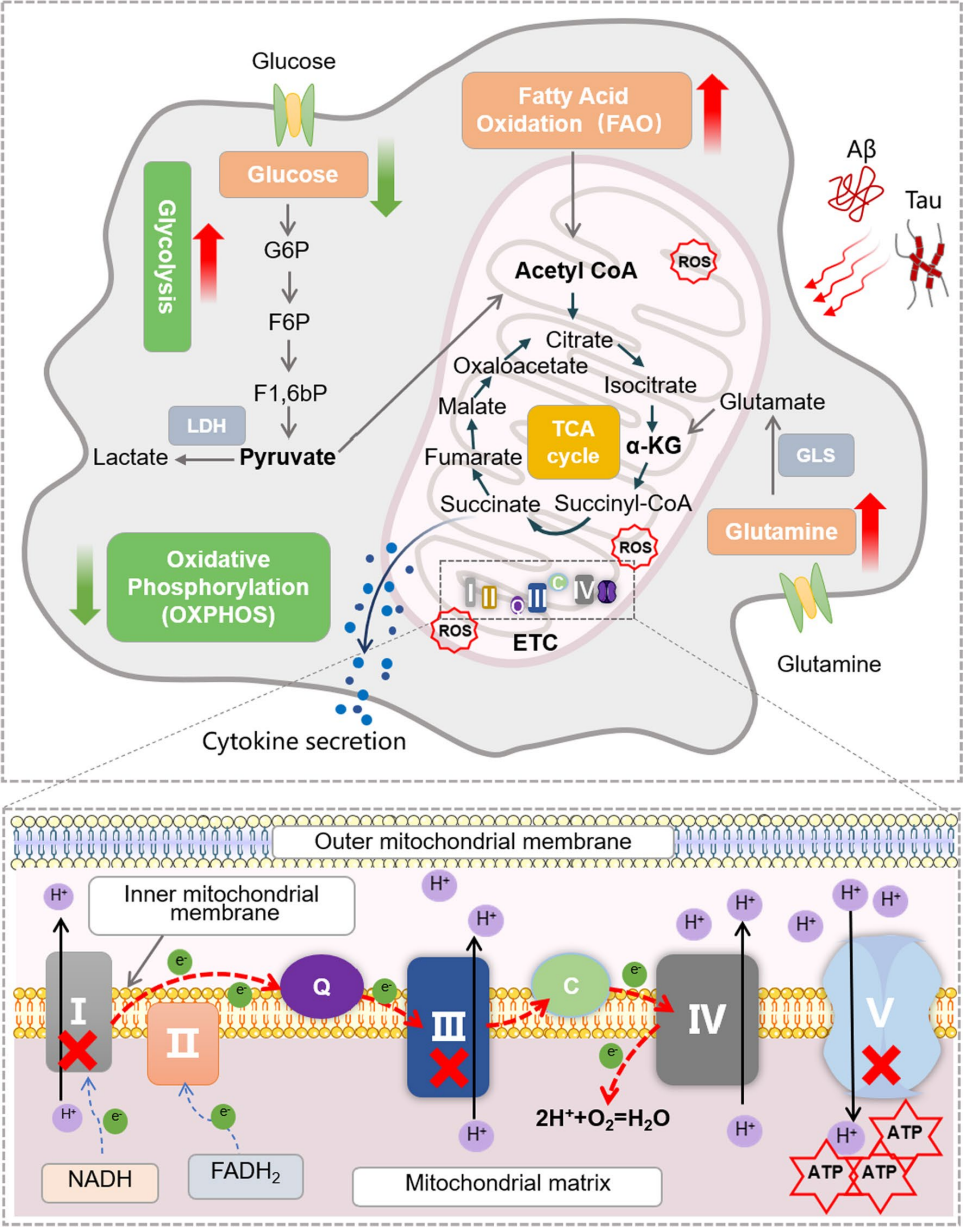
Mitochondrial energy metabolism in microglia. The high energy consumption of microglia to maintain immune functions is regulated by mitochondria. Under normal circumstances, microglia mainly use glucose oxidation for energy supply. First, after being transported into the cell, glucose is converted into pyruvate through the glycolytic pathway. Pyruvate is then converted into acetyl-CoA in the mitochondria, which participates in the TCA cycle and generates a small amount of energy. The subsequent ETC creates a chemical gradient by transferring electrons that powers the production of ATP. When being insulted by pathogenic factors (e.g., Aβ, p-Tau proteins, etc.) or in a state of stress, energy demands of microglia increase. Microglia exert metabolic flexibility to switch from OXPHOS to glycolysis for energy supply. In addition, microglia also use fatty acids and glutamine as alternative energy sources under some conditions

Mitochondria are the energy factories of cells and play a central role in the energy metabolism of the brain. Mitochondrial energy metabolism is a complicated process that consists of various parts including tricarboxylic acid (TCA) cycle and oxidative phosphorylation (OXPHOS)/ETC. In the cytoplasmic matrix, glucose produces pyruvate and ATP through glycolysis. Pyruvate can also enter the mitochondria and be converted into acetyl-CoA, which generates OXPHOS substrates through the tricarboxylic acid cycle (Fig. 2).

The TCA cycle, performed within the matrix of the mitochondrion, consists of eight enzymatic steps that consume and regenerate citrate [52]. The TCA cycle begins with the reaction that combines acetyl-CoA, generated from carbohydrates, fats, and proteins, with oxaloacetate (OAA) to generate citrate. Citrate is converted into isocitrate, and isocitrate is then converted into α-ketoglutarate (α-KG) and subsequently into succinyl-CoA to release two CO_2_ and generate two electron-rich nicotinamide adenine dinucleotide (NADH) molecules. Next, succinyl-CoA is converted into succinate, along with the generation of GTP for ATP production. Succinate is oxidized by dehydrogenase (SDH) to generate fumarate, and flavin adenine dinucleotide (FADH2) for ETC is also generated in this reaction. Afterwards, fumarate is converted into malate and further into OAA that combines with another acetyl-CoA to continue this circular reaction.

Mitochondria are the main sites for OXPHOS and adenosine triphosphate (ATP) synthesis via ETC in cells. ETC is mainly composed by 5 respiratory chain enzymes, complex I to V, located on the inner membrane of mitochondria [53]. Complex I catalyzes the transfer of electrons from NADH to coenzyme Q (CoQ), where CoQ is reduced to ubiquinol (QH_2_). Following electron transfer, protons are pumped from the matrix into the mitochondrial inner membrane presumably through the ubiquinone redox reaction and the conformation and density changes of water in complex I. In complex II, the extra electrons from succinate are transferred to CoQ, and two hydrogen atoms from the TCA cycle are transferred to FAD to generate FADH2. CoQ transfers electrons to complex III and then to complex IV via cytochrome c. In Complex IV, electrons are transferred to oxygen molecules to form water. Finally, complex V synthesizes ATP from adenosine diphosphate (ADP) by utilizing the energy provided by the proton electrochemical gradient [54].

It is worth-noting that metabolic modes of cells can shift between TCA cycle/OXPHOS and glycolysis. Glycolysis consists of ten reactions that are divided into ATP investment and ATP payoff phases, which takes place in the cytosol [55]. In glycolysis, glucose is converted into glucose 6-phosphate (G6P) which is then converted into fructose 6-phosphate (F6P). F6P is further converted into fructose 1,6-bisphosphate (F1,6bP) to generate pyruvate, and pyruvate is then fermented to lactates and other organic acids. Glycolysis is quicker than OXPHOS to generate ATP without the requirement of oxygen, so the glycolytic pathway is preferred for a fast energy supply [55]. On the other hand, glycolysis is much less efficient than the TCA cycle and OXPHOS for energy generation. However, aerobic glycolysis that converts glucose to lactate even with sufficient oxygen supply has been observed in tumor cells since 1920s, which is called Warburg’s effect [31, 56]. Recent studies also demonstrate the shift of energy generation modes from OXPHOS to glycolysis, known as metabolic reprogramming, in many other cells including microglia under physiological and pathological conditions [31, 57].

### Mitochondrial energy metabolism and AD

Substantial evidence shows that impaired metabolism caused by decreased glucose utilization is a prevalent feature of AD in the early stages [21, 58, 59], and mitochondrial dysfunction might play a key role in glucose hypometabolism and energy impairment in AD [20]. A recent large-scale proteomic study of 2000 brains and 400 cerebrospinal fluid samples found that the protein network module related to glucose metabolism merged as a key pathogenic factor in association with AD pathology and cognitive impairment [60]. Gene expression studies further found that 51 members of glycolysis, TCA cycle, OXPHOS and related pathways were significantly downregulated in post-mortem hippocampal samples of AD patients [61, 62]. An in vitro study further revealed impaired OXPHOS in APP C-terminal fragments (APP-CTFs)-accumulated SH-SY5Y neuronal cells [29].

In the TCA cycle, the pyruvate dehydrogenase (PDH) and 2-oxoglutarate dehydrogenase (2-OGDH) complex have been observed to reduce flux in rodent models of aging and AD [63]. Transgenic AD mice also exhibit reduced 2-OGDH complex function [64, 65]. The levels of TCA cycle-derived metabolites (glutamate, glutamine, GABA, and NAA) are significantly reduced in AD and aging animal models, which is consistent with AD pathology [66]. An investigation on the bioenergetic profiles of fibroblasts from late-onset Alzheimer's disease (LOAD) patients and healthy controls demonstrated a shift in energy production to glycolysis in LOAD fibroblasts [67], indicating that impairment in bioenergetic metabolism may be a key mechanism contributing to the risk and pathophysiology of LOAD. In the AD cell model, the accumulation of APP-CTFs significantly reduces mitochondrial complex I activity [29]. A series of PET measurements with [^18^F]BCPP-EF mitochondrial function, [^11^C]PBB3 for tau deposition, and [^11^C] PiB for amyloid deposition further revealed a strong association of mitochondrial complex I abnormalities with p-Tau and clinical symptoms in mild AD [68]. Moreover, impaired activities of ETC complex III and IV have been found in post-mortem cerebral cortex, temporal cortex, and hippocampus of AD patients [69, 70]. The activity impairment of a single or multiple complexes significantly disrupts the function of the respiratory chain, resulting in insufficient energy supply and mitochondria-related disease ultimately [71]. These findings suggest a strong association of mitochondrial metabolism disorder with the pathogenesis of AD.

### Microglial mitochondrial energy metabolism and AD

#### Mitochondrial energy metabolism and microglial activation

The high energy consumption of microglia to maintain immune functions is regulated by mitochondrial energy metabolism. In the brain, glucose is predominantly used for energy production. Glucose meets energy requirements of microglia through glycolysis and OXPHOS [31, 72, 73]. Microglia express glycolysis and oxidative metabolism genes, and can switch between two metabolic programs in response to energy demands in physiological and pathological conditions [31, 72, 73]. In the resting state, microglia mainly rely on OXPHOS to produce ATP. Microglia change from a dormant to a reactive condition in response to inflammation or stress, which is accompanied by partial mitochondrial dysfunction and insufficient energy production. To compensate for the loss of ATP, microglia enhance glucose absorption via GLUT-1 and utilize anaerobic glycolysis for fast energy supply, resulting in enhanced immune responses [74, 75]. Studies revealed that the inhibition of OXPHOS/ETC activity activates microglia [76, 77]. In primary cultured microglia/macrophages and BV-2 cell lines, inhibition of ETC complexes through corresponding inhibitors causes a series of cellular reactions, including alteration of cell morphology, induction of mitochondrial ROS generation-driven oxidative stress, activation of MAPK/NF-κB pathway and NLRP3 inflammasomes, elevation of pro-inflammatory cytokine production, and acceleration of damaged mitochondria accumulation, thereby leading to microglia/macrophage dysfunction and apoptosis [76, 77]. These findings indicate the modulation of microglial immune responses by metabolic reprogramming.

Besides, microglia also express transporters and other genes involved in fatty acid and glutamine metabolism [78], enabling microglia to adapt to various metabolic conditions that regulate microglial polarization status and energy metabolism [79]. Fatty acid oxidation (FAO) accounts for 20% of the brain energy demand [80], and microglia can be fueled by FAO in the absence of glucose [81, 82]. Microglia express higher levels of lipoprotein lipase, which is necessary for fatty acid release from triglycerides [81]. Microglia in aged mice undergo changes in energy metabolism, switching from glycolysis to FAO for energy [82]. The utilization of fatty acids as an energy source may partially support the increased energy demands of activated microglia. At the same time, fatty acids are also considered as signaling molecules that influence the activity of microglia [83].

Another metabolic source of microglia is glutamine, which has a high concentration in the brain. Glutamate, the deaminated product of glutamine, is the major excitatory neurotransmitter in the central nervous system [84]. Glutamine is converted to glutamate by glutaminase (GLS). Glutamate is further processed to α-KG by glutamate dehydrogenase (GDH) and α-KG enters the TCA cycle to support mitochondria metabolism [10]. Microglia express GDH, GLS, glutamine transporters SLC1A5 and SLC38A1, as well as major amino acid transporters, implying an active glutamine metabolic pathway in microglia [81]. In hypoglycemic mouse models and acute hypoglycemic brain slices, microglia rapidly adapt to glutamine as an alternative metabolic fuel in a mTOR-dependent manner to maintain OXPHOS capacity and its immune monitoring function in blood glucose [85]. In primary microglia, glutamine alone as an energy source is sufficient to maintain cell proliferation and phagocytosis [86]. Glutamine supplement restores starvation-induced cellular respiration and viability of microglia, including increased ATP/ADP ratio, enhanced cell viability, activated mTOR signaling pathway, and elevated autophagic activity [87]. This metabolic reprogramming enables microglia to maintain their critical immune surveillance functions even in the presence of glucose starvation and impaired neuronal function [85].

#### Metabolic reprogramming and dysfunction of microglia and AD

**Fig. 3:**
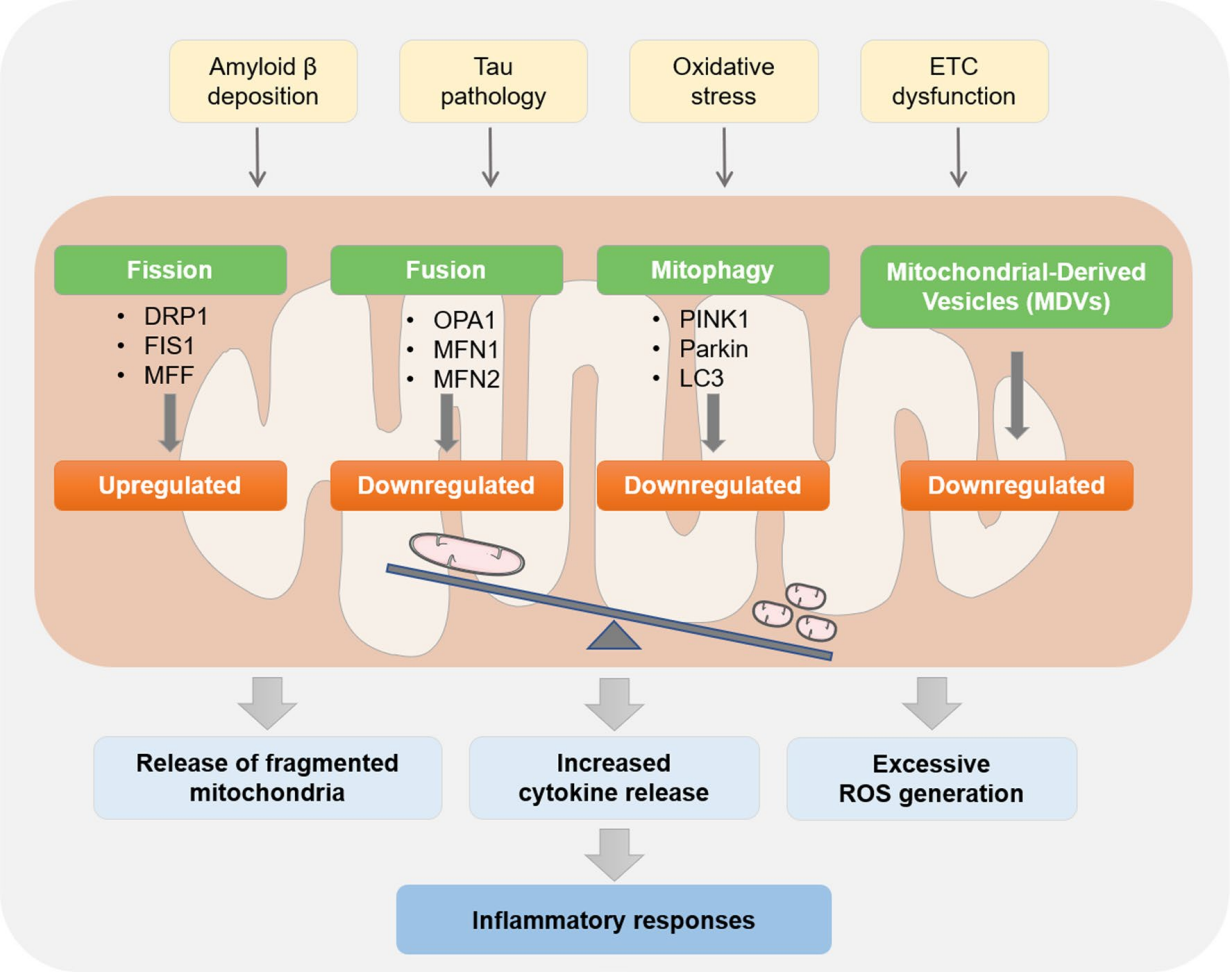
Mitochondrial QC in microglia. Mitochondrial QC is an important process in maintaining microglial homeostasis, mainly including mitochondrial fission, fusion, mitophagy, and MVBs. Microglia in AD are attacked by various pathogenic factors, including Aβ and p-Tau proteins, oxidative stress, ETC dysfunction, etc., which results in an imbalance of mitochondrial QC. The imbalanced microglial mitochondrial QC subsequently causes increased mitochondrial fragmentation, enhanced inflammatory factors release into the extracellular space, and excessive generation of ROS. These pathological changes further exacerbate inflammatory responses of microglia

Microglial mitochondrial energy metabolism disorder and metabolic reprogramming have been reported to be directly linked with the pathogenesis of AD (Fig. 3). High-throughput analyses have implicated that early metabolic changes in the brains of AD patients are associated with microglial activation [60]. Chronic glucose transport impairment has been observed in activated microglia induced by brain damage [88]. The reduced nutrient availability including glucose and altered ETC activity in the pathological conditions of AD impair energy supply, implying oxidative stress damage and metabolic reprogramming of microglia [89].

As expected, metabolic reprogramming was observed in Aβ-stimulated primary microglia [90, 91]. Similarly, microglial metabolic reprogramming was found in specific brain regions of AD mouse models [72], and the brain regions where microglial metabolic reprogramming occurred is consistent with brain regions of Aβ deposition [92]. Following studies demonstrated that Aβ and p-Tau act synergistically to cause mitochondrial toxicity and metabolic dysfunction, characterized by decreased OXPHOS, increased glycolysis, impaired ATP production, interrupted TCA cycle, increased ROS, and accumulated lipid droplets [73, 93]. Metabolic reprogramming and dysfunction induce microglial activation, leading to excessive production of pro-inflammatory cytokines (e.g., IL-1β, TNF-α, and IFN) and neurotoxic molecules that exacerbate neuroinflammation and neurotoxicity in AD [5]. Long-term exposure to Aβ induces the transformation of microglia further into a tolerant state, resulting in defective microglia metabolic systems, diminished inflammatory responses, and impaired Aβ phagocytic capacity [31]. The metabolic disorder in AD is also associated with the deregulation of glutamine/glutamate balance. Abnormal up-regulation of GLS expression has been found in microglia in cell and animal models of AD, which may result in excessive generation of glutamate and interfere microglial metabolic homeostasis [14]. Thus, GLS hyperactivation further induce microglial activation to participate in neuroinflammation in AD, and the treatment of GLS inhibitors JHU-083 or GLS key metabolic intermediate α-KG has been reported to correct microglial metabolic disorder and inflammatory responses, resulting in restoration of cognitive function of AD animal models [14, 94, 95].

To date, various investigations have been carried out to clarify the underlying mechanisms of microglial metabolic reprogramming in AD. In TREM2-deficient AD mice, microglia exhibit accumulated autophagosomes and impaired mammalian target of rapamycin (mTOR) signaling due to down-regulation of energy metabolism [96]. Inhibition of mTOR pathway dramatically reduced Aβ-induced inflammatory responses of microglia through repressing hypoxia-inducible factor-1α (HIF-1α)-dependent glycolysis, whereas boosting glycolytic metabolism by IFN-γ or ATP treatment restored microglial processes motility and increased Aβ phagocytosis [31, 57, 97]. Meanwhile, ATP also induces OXPHOS to correct metabolic reprogramming of microglia [57, 97].

Therefore, these studies suggest an important role of metabolic reprogramming and dysfunction in the regulation of microglial immune functions, including phagocytosis, chemotaxis, cytokine production, membrane biogenesis, and antigen presentation, which contributes to the pathogenesis of AD. An in-depth investigation of the molecular processes that govern microglial metabolism is a critical initial step in our quest to develop effective therapies that target immunometabolism in AD.

## Mitochondrial QC

### QC and homeostasis

Mitochondrial QC is a generic term that includes fission and fusion processes, mitochondrial trafficking, and mitophagy [98]. Mitochondria exist in an ever-changing dynamic state, and mitochondrial fusion and fission are essential for maintaining the growth, shape, distribution, and structure of mitochondria [98].

Mitochondrial fusion is a key step to enhance effective mtDNA and protein distribution in mitochondrial networks [98]. Fusion is coordinated by three different GTPases: optic atrophy 1 (Opa1), mitofusin 1 (Mfn1), and mitofusin 2 (Mfn2). Mfn1 and Mfn2 are localized on the mitochondrial outer membrane, while Opa1 is localized on the inner membrane of mitochondria to maintain normal inner membrane cristae [98]. In contrast, fission promotes the separation of mitochondria containing damaged proteins, unstable membranes, and mutated or damaged mtDNA. The mitochondrial fission process is mainly regulated by dynamin-related protein 1 (Drp1). Drp1 is mainly located in the cytoplasm, but when activated, it is transported to the outer membrane, where it interacts with fission protein 1 (Fis1) and mitochondrial fission factor (Mff) to contract and split mitochondria [99]. The balance of mitochondrial fusion and fission ensures a healthy network of connections, which is critical for the proper positioning of mitochondria to neurons with higher energy demands and the protection of neurons through minimizing oxidative stress [100].

When the contents and organelles of the mitochondria are unavoidably damaged, another two mechanisms of mitochondrial QC are triggered, namely mitophagy and mitochondrial-derived vesicles (MDVs) formation, depending on the degree of damage. When mitochondria are completely dysfunctional, they will be degraded by mitophagy to ensure a homogeneous and healthy mitochondrial population [101, 102]. Mitophagy refers to the encapsulation of depolarized mitochondria into autophagosomes, where they are fused with intracellular lysosomes and degraded, and it plays an important role in cell functions by reducing oxidative stress and restoring homeostasis [101, 102]. PINK1/Parkin is a classical signaling pathway for mitophagy. In damaged and depolarized mitochondria, PINK1 accumulates on the outer mitochondrial membrane and recruits the E3-ubiquitin ligase Parkin [101, 102]. Parkin then labels damaged mitochondria by ubiquitinating mitochondrial outer membrane proteins, such as Mfn1, Mfn2, and VDAC1 [103]. Autophagy receptor P62 is recruited and binds to LC3 to form mitophagosomes. Mitophagosomes then fuse with lysosomes, where damaged mitochondria are degraded and removed [102]. MDVs are another pathway for the transport of mitochondrial components to lysosomes when the mitochondria are mild or partly damaged [104]. MDVs carrying damaged mitochondrial components are isolated from mitochondria and targeted at lysosomes, late endosomes, multivesicular bodies, peroxisomes, or perform extracellular actions [105]. Therefore, mitochondrial fission, fusion, mitophagy, and MDVs are intricately linked, which controls cell fates in the processes of aging and neurodegeneration [106].

### Dysregulation of mitochondrial QC and AD

Abnormal mitochondrial dynamics, especially mitochondrial fragmentation resulting from excessive mitochondrial fission, are early events in AD progression [101, 106]. In postmortem brains of AD patients, the expression levels of Opa-1, Mfn1, and Mfn2 are decreased, while that of Fis1 is significantly increased [107, 108]. Changes in post-translational modification of DLP1 in AD are consistent with increased mitochondrial fragmentation [109]. Afterwards, the Reddy group found that the expression levels of mitochondrial fission genes were significantly elevated, while those of fusion genes declined in immortalized mouse primary hippocampal (HT22) neurons transfected with mutant amyloid precursor protein (mAPP) cDNA in an in vitro AD model [110]. This observation was corroborated by increased mitochondrial numbers and reduced mitochondrial length in mAPP-transfected HT22 cells [110]. Following studies revealed that both Aβ and p-Tau interact with Drp1, with a subsequent increase in free radical production [25, 111]. Consequently, Drp1 and Fis1 were activated, leading to excessive mitochondrial fragmentation, defective transport of mitochondria to synapses, reduced synaptic ATP production, and synaptic dysfunction at last [25, 111].

Moreover, the expression levels of mitophagy genes and proteins were significantly reduced in mAPP-transfected HT22 neurons [110] and in AD transgenic mice [112, 113]. Mitophagy failure was then reported in APP-CTFs-overexpressed SH-SY5Y cells and AD transgenic (3 × TgAD) mice, confirming mitochondrial mitophagy disruption in AD pathological conditions [29]. Similar findings have been reported by Hou et al., that mitophagy is significantly inhibited in AD and mitophagy alteration is strongly associated with early Tau pathology in vivo [114]. The restoration of mitophagy reversed Aβ- and p-Tau-induced synaptic dysfunction and cognitive impairment in various AD models [115–117]. Overall, these findings suggest mitochondrial QC dysfunction as an important contributor to AD pathogenesis and a promising therapeutic target for AD.

### Dysregulation of microglia mitochondrial QC and AD

#### Mitochondria dynamic disorder of microglia and AD

Growing literatures have demonstrated damaged mitochondria in activated microglia due to dysregulation of mitochondrial dynamics in a mouse model of AD [118]. These damaged mitochondria are released into the extracellular space to induce an innate immune response by targeting nearby astrocytes [118]. Consequently, more dysfunctional mitochondria are released, resulting in positive feedback that accelerates neuroinflammation. The abnormal glial activation and inflammatory responses in the brains of AD mice can be further ameliorated through inhibiting excessive mitochondrial fission and fragmentation [119]. This finding is corroborated by in vitro studies that demonstrated the inhibition of mitochondrial fission by the knockdown of Drp1 or the treatment of inhibitors that reduce the activities of NF-κB and MAPK signaling pathways and the production of pro-inflammatory factors in activated microglia [120]. Moreover, natural drugs including echinacoside have also been reported to ameliorate neuroinflammation via suppressing Drp1-dependent mitochondrial fission in microglia [121, 122]. Together, aforementioned literatures have shown significant contribution of mitochondria fusion/fission imbalance to microglia-driven neuroinflammation in AD, indicating the preservation of mitochondrial dynamic balance as an effective therapeutic strategy for treating AD neuroinflammation.

#### Abnormal mitophagy of microglia and AD

Recent reports suggest that impaired mitophagy is a potential contributor to AD. Mitochondrial dysfunction and defective mitophagy have been observed in AD patients’ brain samples, which mediate Aβ and p-Tau pathology [117]. However, conflicting results have been reported that PINK1, LONP1 and LC3 expression were up-regulated in the brains of AD patients and animal models, indicating the hyperactivation of mitophagy in AD [62]. Following studies clarified that neurons play a central role for the enhanced PINK-dependent mitophagy in the AD brains, while glial cells exhibit distinct mitophagy status [62, 117, 123, 124]. In the hippocampal microglia of AD mouse, mitophagy was also reduced by 60%, along with an increase in damaged mitochondria [124]. After being stimulated by Aβ, microglia display defective mitochondria accumulation, which increased cytokine release, inhibited amyloid plaques clearance, and promoted inflammatory responses in the brain [62, 117]. All these pathological changes in microglia were abrogated by the re-activation of mitophagy, leading to the restoration of cognitive and memory function in AD [62, 117]. Taken together, these results suggest mitophagy as a key process that mediates microglial inflammatory responses and phagocytic capacity, making mitophagy a potential therapeutic target for AD.

## Mitochondria: a novel target for AD therapeutics

Given the important roles of mitochondria mtDNA, energy metabolism, and QC in the regulation of microglial function, mitochondrial dysfunction has emerged as a promising therapeutic target of AD. To date, therapeutic strategies/drugs that alleviate mitochondrial dysfunction mainly focus on modulating the Warburg effect, restoring mitochondrial fission/fusion balance, and promoting mitophagy in brain cells, especially microglia.

The Warburg effect of immune cells is a key pathological change of AD, and several compounds that modulate aerobic glycolysis have been applied on AD cell and animal models. 2-deoxy-D-glucose (2-DG), a glucose analog with the 2-hydroxyl group replaced by hydrogen, is a well-known inhibitor of glycolysis since it binds to, but cannot be phosphorylated by, hexokinase [125]. Seven-week-dietary intervention of 2-DG has been reported to promote ketogenesis and maintain alternative mitochondrial bioenergetic pathway in both microglia and neurons, and thus, enhance phagocytic capacity of microglia to decrease Aβ burden and oxidative stress in AD mouse models [125]. Besides 2-DG, dimethyl fumarate (DMF), a derivative of the TCA cycle intermediate fumarate, suppresses aerobic glycolysis via inactivating the catalytic cysteine of the glycolytic enzyme glyceraldehyde 3-phosphate dehydrogenase (GAPDH) in immune cells [126]. DMF treatments have been found to ameliorate cognitive deficits, mitigate tauo-/amyloidopathy, and inhibit microglial oxidative/inflammatory responses presumably through modulating the activities of AMPK/SIRT-1, AKT/CREB/BDNF, AKT/GSK-3β, adiponectin/Adipo1R, and NF-κB/IL-1β/ROS trajectories in AD mouse models [127]. In addition, treatment by mTOR signaling inhibitors rapamycin and metformin also inhibits Aβ-induced microglial metabolic reprogramming and inflammatory responses, and thus, mitigates AD phenotypes [31]. Inspiringly, clinical trials to determine the therapeutic effects of aforementioned compounds on AD were initiated or complected (e.g., ClinicalTrials.gov Identifier: NCT04629495, NCT04200911). Results from these clinical studies will determine the feasibility of utilizing microglial metabolic reprogramming as a target for AD treatment.

Imbalanced mitochondrial fission/fusion is also a potential target of AD therapeutics. P110, a selective inhibitor of mitochondrial fission and fragmentation, significantly improved mitochondrial health and decreased Aβ levels in the brains, and ameliorated cognitive impairment of AD mouse models [119]. Moreover, microglial activation induced by neurotoxic proteins were suppressed by P110 in a mechanism dependent on the inhibition of Drp1-Fis1 interaction. Besides the inhibition of excessive mitochondrial fission, the enhancement of mitochondrial fusion by cannabidiol has also demonstrated encouraging outcomes with regard to neuroinflammation suppression [128]. Cannabidiol enhances the level of mitofusin 2 (Mfn2), a mitochondrial fusion protein, and improves mitochondrial function in microglia, therefore mitigating neuroinflammation-induced cognitive impairment [128].

Another therapeutic strategy is to restore impaired mitophagy in AD. With the help of an in vivo drug screening platform using C. elegans, two potent mitophagy agonists have been identified, namely urolithin A (UA), a small natural compound prominent in pomegranate, and actinonin (AC), an antibiotic that induces mitophagy through mitochondrial ribosomal and RNA decay pathways [117]. In different in vitro AD models, UA and CA, along with two other mitophagy enhancers, tomatidine and nicotinamide riboside, improved HT22 cell OXPHOS and protected cells from A- and p-Tau-induced mitochondrial and synaptic toxicities [115, 116]. Moreover, UA and AC treatment restored impaired mitophagy, facilitated microglial phagocytic efficiency of Aβ, mitigated neuroinflammation, inhibited Tau hyperphosphorylation, and enhanced memory function in mouse models of AD [117]. Similarly, the Fang group developed a screening workflow combining advanced artificial intelligence (AI) and classical wet laboratory approaches to identify multiple novel mitophagy inducers, including kaempferol and rhapontigenin, as potential therapeutics for AD [129]. Both kaempferol and rhapontigenin forestall memory loss and ameliorate Aβ and Tau pathologies in 3 × TgAD mice via increasing microglial phagocytosis and inducing mitophagy [129]. Besides, NAD^+^-boosting compounds, such as nicotinamide riboside (NR) and Olaparib (AZD), significantly reduced Aβ proteotoxicity via inducing mitophagy in Aβ-expressing neuroblastoma cells and AD C. elegans and mouse models [62]. The treatment of melatonin also reversed pathologic phagocytosis of microglia and mitochondrial energy metabolism highly likely through restoration of mitophagy by improving mitophagosome–lysosome fusion via Mcoln1, therefore attenuating Aβ pathology and improving cognition [130]. Notably, there are other mitophagy enhancers, such as Quercetin (Qu), a natural flavonoid, that has displayed promising anti-inflammatory effects on depression and neurodegeneration, implying Qu as a potential therapeutic for AD [131]. These findings support that restoration of mitophagy plays a neuroprotective role in AD presumably through microglia-mediated neuroinflammation and Aβ plaque elimination, making mitophagy a promising therapeutic target for AD treatment [13, 24].

Besides, there are many other proteins, peptides, and peptide mimetics that were identified as mitochondrial targeting systems [132]. The therapeutic outcomes of these molecules in AD are with great scientific research and clinical application value. Thus, comprehensive investigations are urgently needed to further evaluate the therapeutic effect of these mitochondrial function-regulating drugs in different AD models and AD patients if applicable.

## Conclusions and future directions

In summary, multiple hypotheses have been proposed for the pathogenesis of AD, one of the most complicated and progressive neurodegenerative disease, and among them, mitochondrial dysfunction has emerged as a hotspot. Growing evidence have demonstrated the tight association of mitochondrial dysfunction and microglia-driven neuroinflammation. Aβ deposition and other pathological changes damage mtDNA, disturb mitochondrial membrane permeability, alter mitochondrial metabolism and QC, leading to microglial activation and neuroinflammation. Moreover, when severely damaged mitochondria are not properly removed by mitophagy, activated microglia release harmful mitochondrial contents such as ROS and reactive nitrogen into the extracellular environment, which damages surrounding neurons and astrocytes to amplify the inflammatory responses. Therefore, microglial mitochondrial dysfunction-driven neuroinflammation causes neuronal loss and neural circuit disorder, resulting in AD ultimately. Inspiringly, strategies that aim to correct mitochondrial dysfunction in activated microglia have obtained convincing outcomes in vitro and in vivo, indicating mitochondrial dysfunction as a promising target for AD treatment.

Although tremendous progress has been made in microglial mitochondria and AD research, there are many open questions remaining to be answered in the future. First, whether there are abnormal movement of mitochondria in microglia in AD? The Reddy group demonstrated loss of mitochondrial axonal transport as an important cause for synaptic degeneration and cognitive decline in AD [24, 25]. It is interesting to investigate the intracellular localization changes of mitochondria and their biological outcomes when microglia are exposed to Aβ and p-Tau. Second, whether current mitophagy enhancers and other relevant compounds have similar therapeutic effects on AD patients? Due to the paucity of AD animal models that are sufficiently similar to humans, the outcomes of current strategies that aim to restore mitochondrial function in AD on human remain unknown. This point could be addressed by multi-center clinical cohort studies or partially answered utilizing non-human primate modes of AD [133]. Third, is there any approach to deliver drug candidates for restoring mitochondrial function to microglia specifically? One option is to package these candidates into artificial or modified natural nanoparticles for targeted delivery, although the targeting efficiency requires to be further improved [133, 134]. Fourth, is there mitochondrial exchange between microglia and other types of brain cells particular neurons in AD? Recent studies reported transfer of mitochondria from astrocytes to neurons post stroke that participates in neuroprotection and neurorecovery [135]. It is interesting to examine the existence of mitochondria transportation pathway between microglia and neurons, and the biological and pathological functions of this pathway in AD. Hence, more in-depth investigations on microglial mitochondria will provide a novel perspective for the pathogenesis of AD, filling a significant knowledge gap and promoting the development of effective therapeutic interventions and accurate early diagnosis of AD.

## Data Availability

Not applicable.

## Abbreviations

2-DG: 2-Deoxy-d-glucose
2-OGDH: 2-Oxoglutarate dehydrogenase
α-KG: α-Ketoglutarate
Aβ: β-Amyloid
AC: Actinonin
AD: Alzheimer’s disease
AI: Artificial intelligence
APP-CTFs: Amyloid precursor protein C-terminal fragments
ATP: Adenosine triphosphate
AZD: Olaparib
cGAS: Cytosolic cyclic GMP-AMP synthase
CNS: Central nervous system
CoQ: Coenzyme Q
DMF: Dimethyl fumarate
Drp1: Dynamin-related protein 1
ETC: Electron transport chain
FAD: Flavin adenine dinucleotide
FADH2: Flavin adenine dinucleotide
FAO: Fatty acid oxidation
Fis1: Fission protein 1
F6P: Fructose 6-phosphate
F1,6bP: Fructose 1,6-bisphosphate
GABA: γ-Aminobutyric acid
GAPDH: Glyceraldehyde 3-phosphate dehydrogenase
GDH: Glutamate dehydrogenase
GLS: Glutaminase
GLUT-1: Glucose transporter-1
GTP: Guanosine triphosphate
G6P: Glucose 6-phosphate
HIF-1α: Hypoxia-inducible factor-1α
IFN: Interferon
IL-1β: Interleukin-1β
LOAD: Late-onset Alzheimer's disease
LONP1: Lon peptidase 1
LPS: Lipopolysaccharide
mAPP: Mutant amyloid precursor protein
MDVs: Mitochondrial-derived vesicles
Mff: Mitochondrial fission factor
Mfn1: Mitofusin 1
Mfn2: Mitofusin 2
MiWAS: Mitochondrial-wide association study
mtDNA: Mitochondrial DNA
mtDNAcn: Mitochondrial DNA copy number
mTOR: Mammalian target of rapamycin
NAA: *N*-Acetyl aspartate
NADH: Nicotinamide adenine dinucleotide
NFTs: Neurofibrillary tangles
NLRP3: NOD-like receptor family pyrin domain containing 3
NR: Nicotinamide riboside
OAA: Oxaloacetate
Opa1: Optic atrophy 1
OXPHOS: Oxidative phosphorylation
p-Tau: Phosphorylated Tau protein
PDH: Pyruvate dehydrogenase
PINK1: PTEN-induced putative kinase 1
QC: Quality control
QH_2_: Ubiquinol
RAGE: Receptor for advanced glycation end products
ROS: Reactive oxygen species
SDH: Succinate dehydrogenase
SLC1A5: Solute carrier family 1 member 5
SLC38A1: Solute carrier family 38 member 1
SNP: Single nucleotide polymorphism
STING: Stimulator of interferon genes
TCA: Tricarboxylic acid
TLRs: Toll-like receptors
TNF-α: Tumor necrosis factor-α
UA: Urolithin A
VDAC1: Voltage-dependent anion channel protein 1
